# PCL/Si-Doped Multi-Phase Calcium Phosphate Scaffolds Derived from Cuttlefish Bone

**DOI:** 10.3390/ma15093348

**Published:** 2022-05-06

**Authors:** Antonia Ressler, Leonard Bauer, Teodora Prebeg, Maja Ledinski, Irina Hussainova, Inga Urlić, Marica Ivanković, Hrvoje Ivanković

**Affiliations:** 1 Faculty of Chemical Engineering and Technology, University of Zagreb, Marulićev trg 19, 10000 Zagreb, Croatia; lbauer@fkit.hr (L.B.); tprebeg@fkit.hr (T.P.); mivank@fkit.hr (M.I.); hivan@fkit.hr (H.I.); 2 Faculty of Science, University of Zagreb, Horvatovac 102a, 10000 Zagreb, Croatia; maja.ledinski@biol.pmf.hr (M.L.); inga.urlic@biol.pmf.hr (I.U.); 3 Department of Mechanical and Industrial Engineering, Tallinn University of Technology, Ehitajate 5, 19086 Tallinn, Estonia; irina.hussainova@taltech.ee

**Keywords:** biogenic source, biomimetic, bone scaffold, calcium phosphate, calcium silicate, silicon

## Abstract

Increasing attention is focused on developing biomaterials as temporary scaffolds that provide a specific environment and microstructure for bone tissue regeneration. The aim of the present work was to synthesize silicon-doped biomimetic multi-phase composite scaffolds based on bioactive inorganic phases and biocompatible polymers (poly(ε-caprolactone), PCL) using simple and inexpensive methods. Porous multi-phase composite scaffolds from cuttlefish bone were synthesized using a hydrothermal method and were further impregnated with (3-aminopropyl)triethoxysilane 1–4 times, heat-treated (1000 °C) and coated with PCL. The effect of silicon doping and the PCL coating on the microstructure and mechanical and biological properties of the scaffolds has been investigated. Multi-phase scaffolds based on calcium phosphate (hydroxyapatite, *α*-tricalcium phosphate, *β*-tricalcium phosphate) and calcium silicate (wollastonite, larnite, dicalcium silicate) phases were obtained. Elemental mapping revealed homogeneously dispersed silicon throughout the scaffolds, whereas silicon doping increased bovine serum albumin protein adsorption. The highly porous structure of cuttlefish bone was preserved with a composite scaffold porosity of ~78%. A compressive strength of ~1.4 MPa makes the obtained composite scaffolds appropriate for non-load-bearing applications. Cytocompatibility assessment by an MTT assay of human mesenchymal stem cells revealed the non-cytotoxicity of the obtained scaffolds.

## 1. Introduction

Bone regeneration research is attracting increasing attention as the incidence of bone fractures in Europe increases by 28% annually, resulting in an additional economic burden [1]. Bone grafts are essential to repair critical-size bone defects (>2 cm^3^), which do not heal through self-regeneration, or in which a present pathologic process prevents healing [2]. Calcium phosphates, biomaterials mostly studied for bone regeneration, have been used as inorganic phases in a variety of scaffolds to improve osteogenic properties of synthetic biomaterials and promote the bone regeneration [2,3]. The most commonly used calcium phosphates are hydroxyapatite (HAp, Ca_10_(PO_4_)_6_(OH)_2_), *β*-tricalcium phosphate (*β*-TCP, *β*-Ca_3_(PO_4_)_2_) and *α*-tricalcium phosphate (*α*-TCP, *α*-Ca_3_(PO_4_)_2_) [3]. Along with calcium phosphates, calcium silicates (e.g., wollastonite, CaSiO_3_; dicalcium silicate, Ca_2_SiO_4_; larnite, *β*-Ca_2_SiO_4_; tricalcium silicate, Ca_3_SiO_5_) have been examined for potential bone tissue engineering applications (BTE), inspired by the remarkable properties of silicate-based bioactive glasses and glass-ceramics [4,5]. Calcium silicates have shown excellent bioactivity and the ability to promote osteoblast proliferation, induce osteoblast differentiation of stem cells and enhance bone regeneration. A multi-phase system strategy combines the properties of phosphate and silicate-based phases, thus representing an effective pathway for the production of biomaterials with adequate properties (e.g., mechanical properties, surface roughness, bioactivity, biodegradation rate) [5].

In 1970, a study by Carlisle [6] demonstrated the crucial role of silicon in the bone regeneration process, formation and growth. Numerous studies have confirmed the results of the study by Carlisle [6], showing that silicon has significant effects on promoting bone formation, regeneration and vascularization, both in vitro and in vivo [7]. Further, calcium phosphates obtained from biogenic sources (e.g., cuttlefish bone, eggshells, seashells) are better accepted in vivo because of their physiochemical similarity to biological apatite [8]. Calcium phosphates obtained from biogenic sources are substituted with elements (e.g., Sr^2+^, Mg^2+^, Na^+^, K^+^) that play a key role in bone regeneration [9]. To combine properties of HAp derived from a biogenic source and silicon, Kim et al. [10] obtained Si-substituted HAp from a biogenic source (cuttlefish bone), in which cell adhesion of stem cells on the porous scaffold was enhanced compared to the non-substituted HAp. Scaffolds based on Si-substituted HAp demonstrated enhanced cell proliferation, DNA quantity and expression of bone-related genes [10]. However, due to the poor mechanical properties of HAp (e.g., brittleness, fracture toughness), scaffolds based on HAp cannot be used in bone defects where load-bearing scaffolds are required. To overcome low mechanical properties, HAp is often combined with various polymers that provide flexibility to the brittle system [11]. A wide range of natural (collagen, chitosan, gelatin, alginate, poly(hyaluronic acid) and synthetic (poly(glycolic acid) (PGA), poly(L-lactic acid) (PLA), poly(ε-caprolactone) (PCL) polymers that undergo enzymatic and/or hydrolytic degradation have been proposed for biomedical applications [2,11]. Naturally derived polymers showed promising preliminary results; however, concerns about the reproducible degradation characteristics, mechanical properties and large amounts of materials needed for commercial applications resulted in the increased usage of synthetic polymers [2].

The aim of the present work was to synthesize a silicon-doped biomimetic multi-phase composite scaffold based on bioactive inorganic phases and biocompatible polymers (PCL) using simple and inexpensive methods. Porous multi-phase composite scaffolds from cuttlefish bone, synthesized using a hydrothermal method, were impregnated with (3-aminopropyl)triethoxysilane (APTES) and heat-treated at 1000 °C. Then, scaffolds were coated with PCL using the vacuum impregnation technique. The effect of silicon doping and the PCL coating on the microstructure and mechanical and biological properties of the biomimetic scaffolds has been examined.

## 2. Materials and Methods

### 2.1. Scaffold Preparation

#### 2.1.1. Hydrothermal Synthesis of Porous Hydroxyapatite

Hydrothermal synthesis of porous HAp was prepared as previously described in our research [11]. In brief, cuttlefish bones were cut into pieces of ~2 cm^3^ and immersed in the solution of sodium hypochlorite (NaClO, 13% active chlorine, Gram-mol) for 24 h. The cuttlefish bone pieces were then washed several times with distilled water (100 °C) and dried at 105 °C. After drying, cuttlefish bone pieces were sealed with the required volume and concentration of ammonium dihydrogen phosphate (NH_4_H_2_PO_4_, 99% Scharlau, Sentmenat, Spain) aqueous solution in TEFLON-lined stainless steel pressure vessel at 200 °C for 48 h. After hydrothermal treatment, HAp pieces were washed with distilled water (100 °C) and dried at 105 °C.

#### 2.1.2. Doping Hydroxyapatite Structure with Silicon

HAp scaffolds obtained from cuttlefish bone were impregnated with APTES (99%, Sigma Aldrich, St. Louis, MO, USA) for 10 min using BD Vacutainer. After impregnation, scaffolds were dried at 110 °C for 24 h and then heat-treated at 1000 °C for 4 h in order to remove the organic part. The same procedure was repeated for 4 cycles. The samples were designated as CaP_Si0, CaP_Si1, CaP_Si2, CaP_Si3 and CaP_Si4, where the number denotes the number of repeated procedures.

#### 2.1.3. Preparation of PCL/CaP_Si Scaffolds

**Figure 1:**
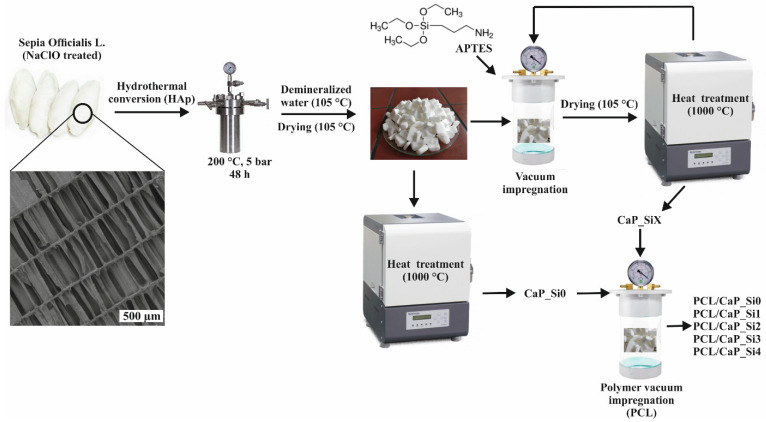
Schematic illustration of PCL/CaP_Si scaffold preparation. The first step involves the hydrothermal conversion of the cuttlefish bone aragonite structure into hydroxyapatite scaffolds, followed by impregnation with APTES and heat treatment. The final step includes coating bioactive scaffolds with PCL solution to produce porous PCL/CaP_Si composites.

CaP_Si scaffolds were impregnated with 10 *w*/*v* % PCL (M_n_ = 45,000, Sigma Aldrich) dissolved in the chloroform (CHCl_3_, p.a. Kemika, Ovada, Italy) using the vacuum impregnation unit (CitoVac, Struers, Cleveland, OH, USA) as previously described in our research [11]. In brief, the porous CaP_Si scaffolds were put in a glass and placed in the vacuum chamber under a pressure of 0.11 bars. After 10 min at 0.11 bars, scaffolds were soaked in PCL solution for an additional 10 min. After impregnation, soaked CaP_Si scaffolds were placed on cotton fabric and then in the vacuum chamber at 0.11 bars for 10 min. The process was repeated in order to remove excess PCL solution and to dry the scaffolds. The composite scaffold preparation procedure is illustrated in Figure 1.

### 2.2. XRD Analysis and Whole-Powder-Pattern Decomposition Refinement

The prepared CaP scaffolds treated with APTES–heating cycles were mixed with silicon standard (5 wt%, NIST SRN 640e, Sigma Aldrich) and were characterized by X-ray diffraction (XRD) analysis (Shimadzu XRD-6000, Kyoto, Japan) with Cu K_α_ (1.5406 Å) radiation operated at 30 mA and 40 kV, in the 2*θ* range 20°–60° with a step size of 0.02° and exposure of 5 s. Identification of the crystalline phases was performed by comparing the experimental XRD patterns with standards by the International Centre for Diffraction Data (ICDD) using the card 09-432 for HAp, 09-0348 for *α*-tricalcium phosphate (*α*-TCP), 70–2064 for whitlockite (Wtc), 42-550 for wollastonite (CaSiO_3_), 05-0586 for dicalcium silicate (Ca_2_SiO_4_), 09–351 for larnite (*β*-Ca_2_SiO_4_) and 82–1691 for calcium oxide (CaO).

Whole-powder-pattern decomposition refinement studies were performed as previously described in our studies [9] using the software DIFFRAC.SUITE TOPAS V.5.0. with the fundamental parameters approach. In brief, the structural parameters of HAp reported by Veselinović et al. [12], *α*-TCP by Mathew et al. [13], whitlockite by Zatovsky et al. [14], CaSiO_3_ by Ohashi [15], Ca_2_SiO_4_ by Toraya and Yamazaki [16], *β*-Ca_2_SiO_4_ by Tsurumi et al. [17] and CaO by Primak et al. [18] were used as the initial values in the refinements. Refined parameters were specimen displacement, phase weight percentage and scale factor. The weighted profile R factor (R_wp_ < 10%) and expected R factor (R_exp_ < 3%) were used to assess the goodness of fit of the refinements.

### 2.3. SEM-EDS Analysis

The morphology of prepared scaffolds before and after coating with the PCL was imaged by the scanning electron microscope (SEM, Zeiss EVO MA 15, Oberkochen, Germany) at an electron beam energy of 10 keV. Prior to analysis, samples were sputter-coated with gold and palladium for 120 s. For a more detailed analysis, energy-dispersive X-ray spectroscopy (EDS) was performed at an electron beam energy of 20 kV in order to examine trace elements and elemental distribution in CaP_Si scaffolds.

### 2.4. Porosity

The porosity of the scaffolds was evaluated according to our previous study [9]. In brief, the PCL/CaP_Si scaffolds (n = 5) were cut into cylindrical pieces 6 mm in diameter (*D*) and ~1 mm in thickness (*H*). Archimedes’ principle was employed by immersing PCL/CaP_Si scaffolds in ethanol (*ρ* = 0.789 g cm^−3^) at room temperature. The scaffolds’ porosity (%) was calculated as a fraction of the pore volume (*V*_pore_) and total volume of the PCL/CaP scaffold (*V*_PCL/CaP_) according to Equation (1):

(1) $$\mathrm{Porosity}\;\left(\%\right)=\frac{V_{\mathrm{pore}}}{V_{\mathrm{PCL}/\mathrm{CaP}}}$$

The pore volume was calculated according to Equation (2) where initially weighted dry scaffolds are represented as *W_d_* and samples weighted after immersion in ethanol are represented as *W_e_*:

(2) $$V_{\mathrm{pore}}=\frac{W_{e}-W_{d}}{\rho_{\mathrm{ethanol}}}$$

The density of the PCL/CaP_Si cylindrically shaped scaffold was calculated according to Equation (3):

(3) $$\rho_{\mathrm{PCL}/\mathrm{CaP}}=\frac{W_{d}}{\pi \;\cdot {\left(\frac{D}{2}\right)}^{2\;}\cdot \;H}$$

### 2.5. FTIR Analysis

Attenuated total reflectance (ATR) spectrometer for solids with diamond crystal (Bruker Vertex 70, Billerica, MA, USA) was used for the Fourier transform infrared spectra (FTIR) analysis. The analysis was performed at room temperature (20 °C) over the spectral range of 4000–400 cm^−1^, with 32 scans and a 4 cm^−1^ resolution for all prepared scaffolds before and after coating with PCL.

### 2.6. Compression Tests

The compression test was carried out by a servo-hydraulic model 8500 universal testing machine (Instron Itd., High Wycombe, UK) with a 500 N maximum load at a crosshead speed of 0.4 mm min^−1^ in ambient conditions. The cube blocks (4 × 4 × 4 mm, n = 5) of prepared CaP_Si and PCL/CaP_Si porous scaffolds were exposed to compressive loads perpendicular to the lamellae of each scaffold. Obtained stress–strain curves were used to determine the compressive strength.

### 2.7. Thermal Analysis

Thermogravimetric analysis (TGA, Netzsch STA 409, Netzsch Instruments, Selb, Germany) was performed with a constant synthetic air flow of 30 cm^3^ min^−1^ from 40 °C to 1200 °C at a heating rate of 10 °C min^−1^ for composite PCL/CaP_Si scaffolds.

### 2.8. Protein Adsorption Assay of Doped CaP Powders against BSA

The BSA protein adsorption on scaffolds’ powders was obtained according to the protocol reported by Shi et al. [19]. In brief, 10 mg of powders (CaP_Si0, CaP_Si1, CaP_Si2, CaP_Si3 and CaP_Si4) were incubated with 1 mL of bovine serum albumin (BSA, Sigma Aldrich, ≥96%) protein (250 μg/mL, protein/phosphate-buffered saline) in triplicates for 4 h at 37 °C. Following the incubation, protein concentration of the supernatant was measured using the BCA assay (Santa Cruz Biotechnology, Dallas, TX, USA). Adsorbed protein concentration was calculated after subtracting the protein concentration of the supernatant (non-adsorbed protein) from the initial BSA concentration. The results are presented as the percentage (%) of adsorbed protein in relation to the negative control.

### 2.9. Cytocompatibility Assay

#### 2.9.1. Preparation of Extracts of CaP Powders

Following the sterilization under ultraviolet light (15 min), CaP_Si powders were soaked in high-glucose Dulbecco’s modified Eagle’s culture medium (DMEM, Sigma-Aldrich), supplemented with 10% fetal bovine serum (FBS, Capricorn Scientific, Ebsdorfergrund, Germany) and 1% penicillin–streptomycin (Capricorn Scientific), at a concentration of 10 mg/mL. After incubation at 4 °C for 24 h, CaP_Si powders suspensions were centrifuged at 300× *g* for 5 min in order to spin down the powders.

#### 2.9.2. Human Mesenchymal Stem Cells Culture

Human mesenchymal stem cells (hMSC) were kindly provided by prof. Inga Urlić, Faculty of Science, University of Zagreb. They were isolated from bone marrow aspirates and propagated as previously reported [20]. hMSC were cultured in low-glucose DMEM (1 g/L) supplemented with 10% FBS (Capricorn Scientific), 1% penicillin–streptomycin and 10 ng mL^−1^ human fibroblast growth factor 2 (FGF2, Gibco, Thermo Fisher Scientific, Waltham, MA, USA).

#### 2.9.3. MTT Assay

Viability of hMSC cells, after 1 and 3 days of treatment with DMEM extracts of the CaP_Si powders, was assessed by using MTT assay (Sigma Aldrich) as previously described in our studies [9,21]. In brief, the hMSC cells were seeded in a 96-well plate (Sarstedt, Nümbrecht, Germany) at a density of 5 × 10^4^ cells and were allowed to adhere overnight at 37 °C, 5% CO_2_. The next day, cell culture medium was removed and cells were treated with DMEM extracts of the CaP_Si powders. Following the incubation period, medium was removed and 40 μL of MTT solution (0.5 mg mL^−1^) was added to each well. After 3 h of incubation at 37 °C, 170 μL of dimethyl sulfoxide (DMSO, Sigma Aldrich) was added to each well to dissolve formazan crystals. The solution was set for colorimetric detection at 560 nm using a microplate reader (GlowMax-Multi, Promega, Madison, WI, USA). The results are presented as a percentage of cell viability and calculated in reference to negative control (untreated cells).

### 2.10. Statistical Analysis

The results of mechanical and biological analysis are presented as the mean ± standard deviation and were analyzed with Student’s *t*-test, in which differences were considered statistically significant when *p* < 0.05.

## 3. Results

### 3.1. Mineralogical Phase Composition

Three-dimensional HAp scaffolds were obtained by hydrothermal conversion of cuttlefish bone (CaCO_3_) in the presence of PO_4_^3−^ according to Equation (4) [8]:

10CaCO_3_ + 6NH_4_H_2_PO_4_ + 2H_2_O → Ca_10_(PO_4_)_6_(OH)_2_ + 3(NH_4_)_2_CO_3_ + 7H_2_CO_3_ (4)

**Figure 2:**
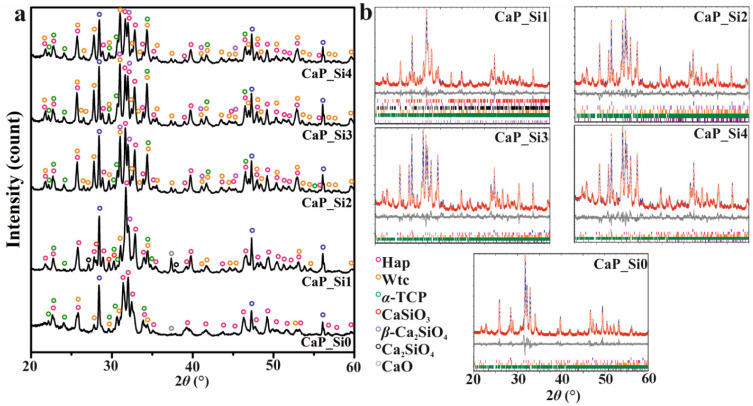
X-ray diffraction data of the prepared calcium phosphate scaffolds doped with silicon (**a**). Whole-powder-pattern decomposition analysis of scaffolds’ X-ray diffraction data (**b**). The solid lines (blue) are calculated data intensities, and open circles are (red) experimental data intensities. The difference between the calculated and experimental intensities is plotted below the XRD profile. Bragg positions of HAp (pink), Wtc (orange), *α*-TCP (green), CaSiO_3_ (red), *β*-Ca_2_SiO_4_ (purple), Ca_2_SiO_4_ (black), CaO (gray) and silicon (blue, standard) are marked for each pattern.

Obtained HAp scaffolds were treated with APTES and heated at 1000 °C as schematically described in Figure 1. The XRD patterns of prepared scaffolds are shown in Figure 2a. Comparing the experimental diffraction pattern to ICDD standards, the crystalline phases were ascribed to HAp, Wtc, *α*-TCP, CaSiO_3_, *β*-Ca_2_SiO_4_, Ca_2_SiO_4_ and CaO, indicating the formation of multi-phase scaffolds based on calcium phosphate and calcium silicate phases. However, due to the formation of the multi-phase system and overlapping of the peaks, it was hard to distinguish peaks between crystalline phases. Therefore, the whole-powder-pattern decomposition refinement analysis of the powder XRD patterns was performed as shown in Figure 2b. The results of quantitative phase analysis are shown in Table 1, indicating a significant amount of an amorphous/glassy phase in all prepared samples treated with APTES. The lowest amount of the amorphous/glassy phase, 6 wt%, was detected in the CaP_Si0 sample that was not treated with APTES. After the first APTES and heat treatment, the amorphous phase increased to 28 wt%, while with further treatments the amount of the amorphous/glassy phase in the range from 35 to 39 wt% was estimated. As the number of APTES treatments and heat treatment cycles increased, the transformation of HAp to Wtc and *α*-TCP was more pronounced, while the total weight percentage of all calcium silicate phases decreased, indicating the additional formation of the glassy phase.

### 3.2. Element Content and Protein Adsorption

**Figure 3:**
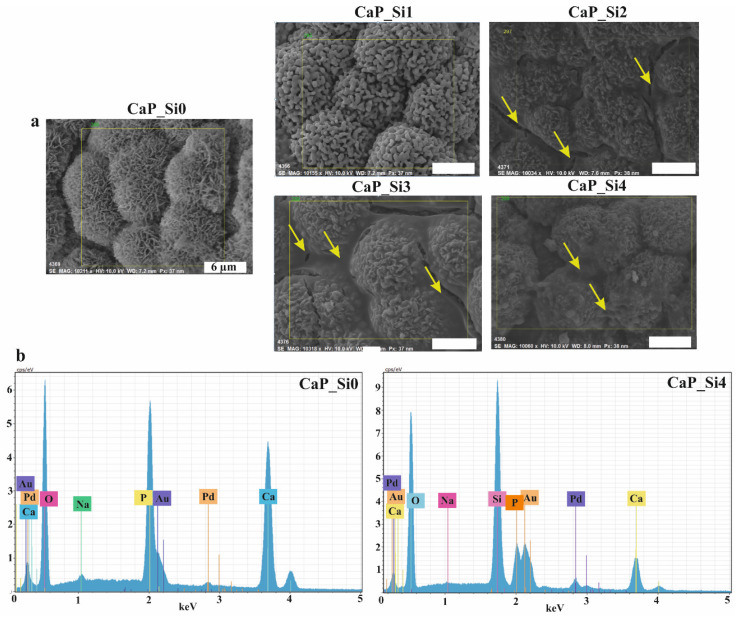
(**a**) SEM micrographs of CaP crystals and (**b**) EDS spectrum of the CaP_Si0 and CaP_Si4 scaffolds. Glassy phase between globules is depicted by yellow arrows. Scale bar: 6 μm.

SEM images (Figure 3a) of observed areas indicate the existence of dandelion-like crystals forming cauliflower-shaped morphology in the CaP_Si0 scaffold. Slightly different morphology with irregular spherical or elongated grains was observed in the CaP_Si1 scaffold. The glassy layer on the obtained crystalline phases was evident in the samples CaP_Si2, CaP_Si3 and CaP_Si4. The glassy phase was more evident between the globules in samples CaP_Si2, CaP_Si3 and CaP_Si4 (represented by yellow arrows). The EDS analysis of formed phases was used to confirm the atomic composition of prepared scaffolds. EDS spectra of the CaP_Si0 and CaP_Si4 scaffolds are compared in Figure 3b. EDS of the CaP_Si0 scaffold revealed the presence of sodium (Na) along with calcium (Ca) and phosphorus (P). In our previous study [21], the chemical composition of HAp obtained from cuttlefish bone was determined by inductively coupled plasma mass spectrometry (ICP-MS), and the presence of Na^+^, Sr^2+^ and Mg^2+^ ions was detected. The HAp obtained from cuttlefish bone was substituted with 2.80 mol% of Na^+^, 0.49 mol% of Sr^2+^ and 0.89 mol% of Mg^2+^ ions. A possible reason for why EDS did not detect strontium and magnesium is their lower concentrations compared to the sodium. The EDS spectra of all CaP_Si scaffolds revealed the presence of the silicon.

**Figure 4:**
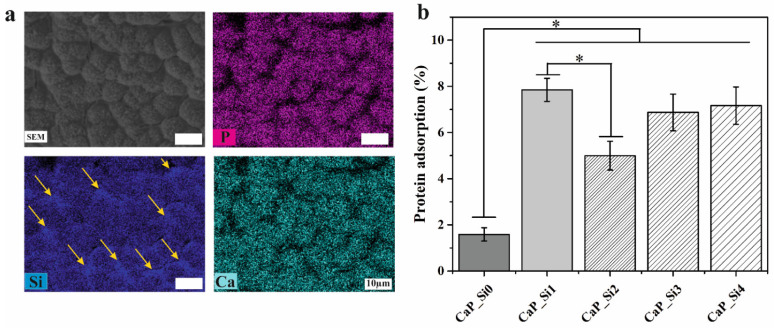
(**a**) EDS elemental mapping of phosphorus (P), silicon (Si) and calcium (Ca) of the sample CaP_Si4. Scale bar: 10 μm. (**b**) The protein adsorption (%) of BSA CaP_Si powders. Silicon between globules is depicted by yellow arrows. The significant difference between the two groups (*): *p* < 0.05.

In addition, EDS mapping (Figure 4a) indicated that calcium and phosphorus were uniformly distributed over the entire investigated area. However, silicon was more concentrated on the surface and between globules (represented by yellow arrows), where the formation of the glassy phase was noticed (Figure 3a). Further, BSA protein adsorption on all prepared CaP_Si powders was performed in phosphate-buffered saline at 37 °C (pH 7.4), and results are shown in Figure 4b. The difference in powder protein adsorption capacity is related to the effect of surface charges and the surface area [19]. It is obvious that Si-doped powders exhibit higher protein adsorption compared to non-treated CaP powders. However, the BSA protein adsorption was not increased with the higher number of treatments with APTES.

### 3.3. Microstructure and Porosity

**Figure 5:**
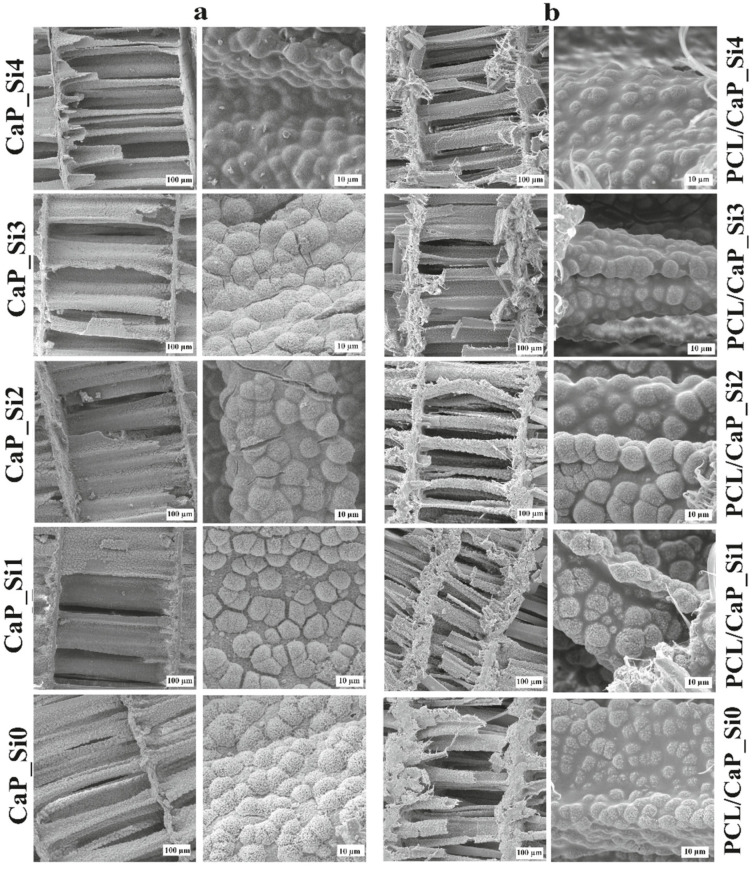
SEM micrographs of calcium phosphate scaffolds doped with silicon before (**a**) and after (**b**) impregnation with PCL. Scale bar: 100 and 10 μm.

SEM micrographs of scaffolds doped with silicon before (a) and after (b) impregnation with PCL are shown in Figure 5. As previously confirmed, the hydrothermal conversion into HAp retains cuttlefish bone’s original porous microstructure, which was also maintained after APTES and heat treatment. Irregularly shaped microspheres on the surface of lamellae and pillars enhanced the surface roughness and surface area. Compared to the scaffold CaP_Si0, scaffolds CaP_Si2, CaP_Si3 and CaP_Si4 show decreased roughness and a smoother surface due to the glassy layer. Micro-cracks were evident in the CaP_Si2 and CaP_Si3 samples, which might be a result of thermal stresses in the glassy layer during the heat treatment.

After polymer (PCL) impregnation, the high porosity and interconnectivity of pores in all prepared scaffolds were maintained. PCL covered calcium phosphate and calcium silicate polycrystalline structures, resulting in the decreased roughness and smoother surface of PCL/CaP_Si composite scaffolds. However, the roughness of cauliflower-like crystals was still present in the samples PCL/CaP_Si0, PCL/CaP_Si1 and PCL/CaP_Si2, whereas samples PCL/CaP_Si3 and PCL/CaP_Si4 showed lower roughness. The determined porosity was 81.94 ± 3.21% in PCL/CaP_Si0, 75.90 ± 3.76% in PCL/CaP_Si1, 76.71 ± 7.43% in PCL/CaP_Si2, 78.59 ± 8.29% in PCL/CaP_Si3 and 76.14 ± 3.36% in PCL/CaP_Si4. After the first treatment with APTES, the porosity decreased, but further treatments with APTES caused no significant difference in porosity.

### 3.4. FTIR Spectra of CaP_Si and PCL/CaP_Si Scaffolds

**Figure 6:**
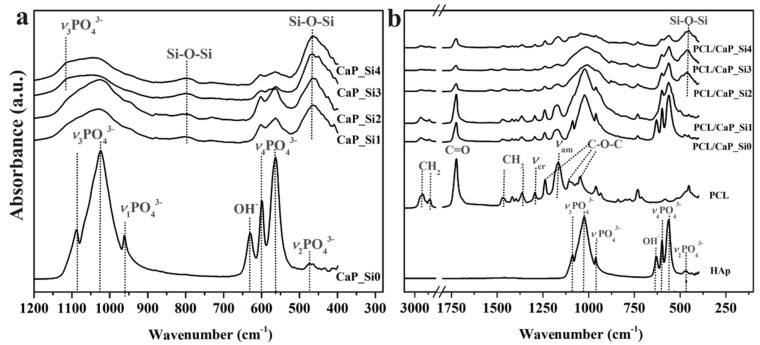
FTIR spectra of CaP_Si (**a**) and PCL/CaP_Si (**b**) scaffolds.

To further characterize CaP_Si (Figure 6a) and the composite PCL/CaP_Si (Figure 6b) scaffolds, FTIR spectroscopy was performed. FTIR spectra (Figure 6a) are shown in the range 400–1200 cm^−1^, while at the wavenumbers >1200 cm^−1^, significant bands were not detected. In the CaP_Si0 scaffold, typical bands of the phosphate (PO_4_^3−^) group at 1024 and 1089 cm^−1^ (asymmetric stretching vibration of P–O), 560 and 603 cm^−1^ (asymmetric bending vibrations of O–P–O) and 962 cm^−1^ (symmetric stretching vibration of P–O) can be assigned to HAp. The band at 473 cm^−1^ was assigned to the *ν*_2_ domain of the PO_4_ thetraedar. The absorption bending vibrations at ~634 cm^−1^ are characteristic of the structural OH– group in HAp crystal [11,22]. After APTES and heat treatment, significant changes were observed in FTIR spectra. CaP_Si1 and CaP_Si2 showed similar spectra, where, along with characteristic bonds of P–O of HAp at 560 and 603 cm^−1^, the shoulders at ~1060 cm^−1^ evidently correspond to HAp, Wtc and *α*-TCP. With additional APTES treatments, shoulders at ~1060 cm^−1^ were broader and the intensity of the characteristic bands of P–O of HAp at 560 cm^−1^ and 603 cm^−1^ decreased. In addition, the results indicate that APTES treatment led to the formation of a glassy silicate layer on the surface of the formed calcium phosphates and calcium silicates. Bonds at 469 cm^−1^ and 795 cm^−1^ correspond to Si–O–Si stretching characteristic of formed silicate glass networks. The bands in the regions 1089–1095 cm^−1^ and 958–962 cm^−1^ can be attributed to P–O/Si–O stretching due to the presence of calcium phosphates and calcium silicates [23].

The FTIR spectra of PCL/CaP_Si composite scaffolds were compared with spectra of the PCL- and non-treated sample (mainly HAp) to identify interactions between PCL and bioactive components. The composite scaffolds showed previously mentioned P–O and Si–O–Si bands characteristic of calcium phosphates and the glassy silicate layer. The PCL spectrum showed characteristic peaks of CH_2_ bending modes at 1473, 1397 and 1361 cm^−1^, CH_2_ stretching at 2942 and 2862 cm^−1^, C=O stretching vibrations at 1726 cm^−1^ and C–O–C stretching vibrations at 1233, 1107 and 1042 cm^−1^. The bands at 1160 and 1290 cm^−1^ were ascribed to C–O stretching in the amorphous phase and C–C stretching in the crystalline phase of PCL [11,24]. In the composite scaffolds, no other bands or bands shift of PCL or bioactive phases were observed, indicating that no chemical reactions occurred between components.

### 3.5. Mechanical Properties

**Figure 7:**
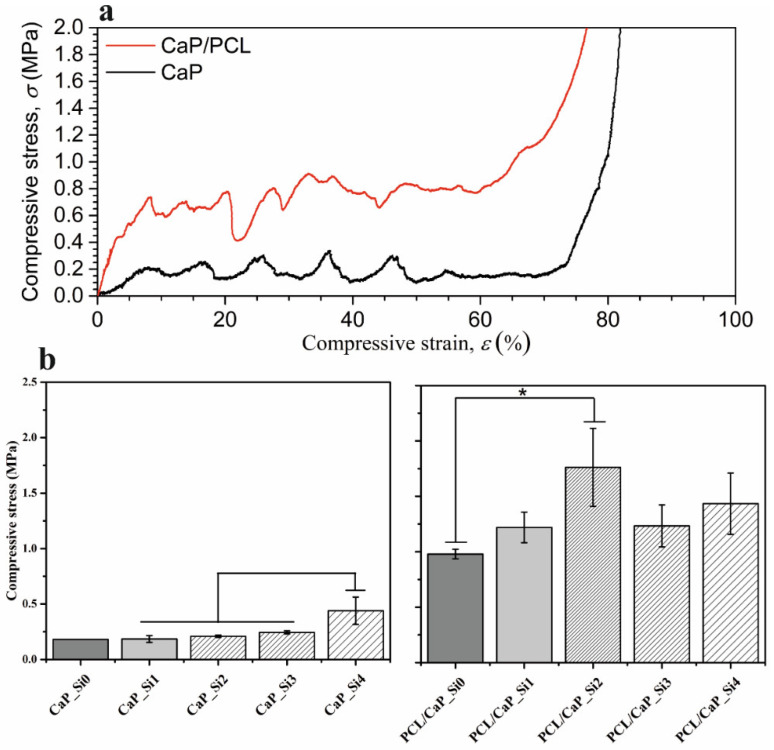
Typical compressive stress–strain curves for CaP_Si0 and composite PCL/CaP_Si0 scaffolds (**a**). Compressive stress of all prepared scaffolds (**b**). The significant difference between two groups (*): *p* < 0.05.

To determine the effect of APTES treatment and PCL impregnation on the mechanical properties of scaffolds obtained from cuttlefish bone, compression tests were performed on CaP_Si and PCL/CaP_Si scaffolds. The compressive stress–strain curves for uncoated (CaP_Si0) and coated (PCL/CaP_Si0) specimens are compared in Figure 7a, showing three different regions typical of porous structures, such as scaffolds derived from cuttlefish bone [11,25]. As the linear force was applied, the initial increase in the stress with low strain (linear elastic region) was present (Zone 1). Further compression application resulted in a multi-peak profile with sawtooth behavior (Zone 2) attributed to the layer-by-layer collapse of the microstructure. Terminal load increase caused a densification region (Zone 3) characterized by a steep increase in stress, when the initially porous specimens crumbled into compact powder. The maximum stress from the linear elastic region was used to quantify the compressive strength of the porous scaffolds, and the results for all prepared samples are shown in Figure 7b. There was no significant difference in the compressive stress of scaffolds CaP_Si0 (0.18 MPa), CaP_Si1 (0.19 MPa), CaP_Si2 (0.21 MPa) or CaP_Si3 (0.24 MPa); however, a significant increase was observed for the CaP_Si4 scaffold (0.44 MPa). PCL coating was effective at increasing mechanical properties of the CaP_Si scaffolds as the compressive strength of PCL/CaP_Si1 (1.22 MPa) increased without a significant difference compared to the PCL/CaP_Si0 (1.22 MPa) scaffold. A significant increase was determined for PCL/CaP_Si2 (1.76 MPa), PCL/CaP_Si3 (1.23 MPa) and PCL/CaP_Si4 (1.43 MPa) scaffolds compared to PCL/CaP_Si0.

### 3.6. Thermogravimetric Analysis of PCL/CaP_Si Scaffolds

**Figure 8:**
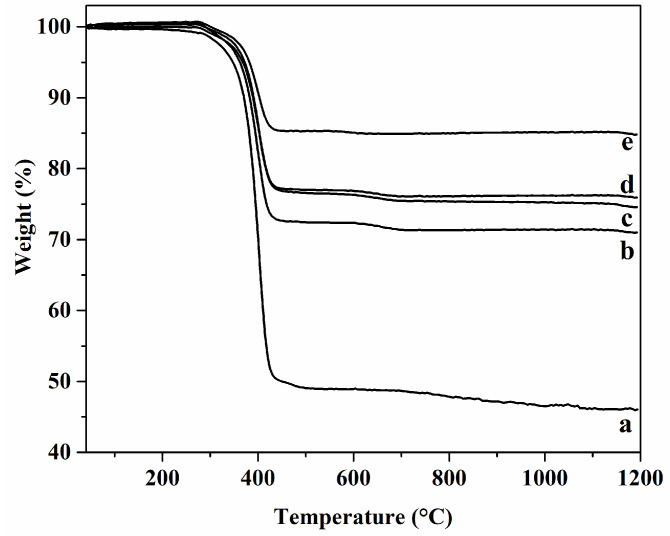
Results of thermogravimetric analysis of composite samples (a: PCL/CaP_Si0; b: PCL/CaP_Si1, c: PCL/CaP_Si2, d: PCL/CaP_Si3, e: PCL/CaP_Si4).

Thermogravimetric analysis was performed to determine the amount of the PCL in the prepared composite scaffolds (Figure 8). Weight loss of the PCL/CaP_Si composite scaffold until 550 °C can be attributed to the PCL degradation. The results show that composite scaffolds PCL/CaP_Si0, PCL/CaP_Si1, PCL/CaP_Si2, PCL/CaP_Si3 and PCL/CaP_Si4 contained 41.1 wt%, 28.6 wt%, 24.9 wt%, 23.8 wt% and 14.8 wt% of PCL. It seems that the dandelion-like structure and cauliflower-like morphology on the surface of the CaP_Si0 scaffold provided an efficient bonding of the PCL coating to the inorganic scaffold. The observed decrease in the PCL content in the scaffolds as the number of APTES treatments increased may be due to the decreased roughness and smoother surface as a result of the formed glassy Si–O–Si layer on the surface of the CaP_Si scaffolds. However, although the PCL/CaP_Si0 scaffold had a significantly higher amount of PCL, the observed mechanical properties were the lowest compared to other prepared composite scaffolds, which may be due to the PCL/CaP_Si0 scaffold having the highest porosity.

### 3.7. Cytocompatibility Assesment of the CaP_Si Scaffolds

**Figure 9:**
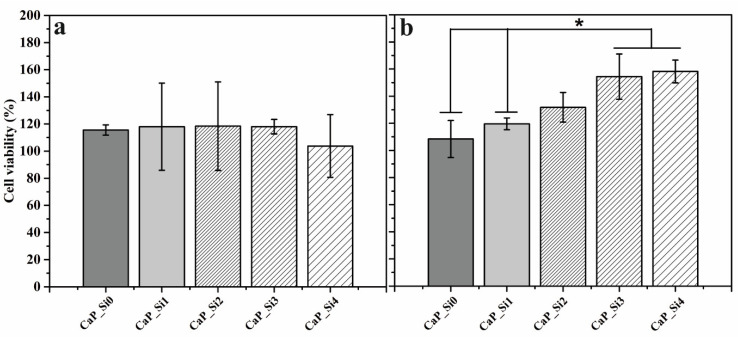
Cell viability (%) after (**a**) 1 and (**b**) 3 days of incubation with DMEM extracts of calcium phosphate powders determined by MTT assay. The significant difference between the two groups (*): *p* < 0.05.

The MTT assay was performed on hMSC 1 and 3 days after treatment with extracts of CaP_Si0, CaP_Si1, CaP_Si2, CaP_Si3 and CaP_Si4 scaffolds (Figure 9). The results are expressed as cell viability (%). According to the ISO 10993-5:2009, the materials are considered non-cytotoxic when cell viability is ≥70%. After 1 day of treatment, there was no significant difference in cell viability between prepared samples. However, after 3 days of treatment, the cell viability was significantly higher for cells incubated in extracts of CaP_Si3 and CaP_Si4 compared to those incubated in the CaP_Si0 and CaP_Si1 scaffolds, while those incubated in the extract of the CaP_Si2 scaffold increasing without a significant difference. The CaP_Si0 and CaP_Si1 scaffolds contained 0.58 and 1.10 wt% CaO, respectively. However, the presence of CaO in the scaffold did not have any negative effect on the cell viability, and cell viability was higher compared to the non-treated cells. The obtained results indicate no toxic effect and excellent biocompatibility of the investigated CaP_Si scaffolds.

## 4. Discussion

An increasing number of studies are focused on developing biomaterials as scaffolds that provide a specific environment and microstructure during the bone regeneration process [26]. In this study, a biomimetic approach was applied for scaffold development where multi-phase bioactive materials were obtained through hydrothermal conversion of cuttlefish bone, preserving its original highly porous structure. Rogina et al. [27] synthesized an HAp-based scaffold from cuttlefish bone and determined channel-like pores with an average height of 200–300 μm and an average width of 40–60 μm. In vivo studies by Oh et al. [28] showed that a scaffold with a pore size ranging from 290 to 310 μm is optimal for efficient bone regeneration. Therefore, the pore size of scaffolds obtained from cuttlefish bone is suitable for efficient bone regeneration. Along with proper interconnectivity between the pores, essential for the diffusion of nutrients, metabolic waste and oxygen, the scaffold porosity should be in the range of 50–90% [29].

After the hydrothermal conversion of aragonite cuttlefish bone structure to HAp, porous scaffolds were impregnated with APTES and heat-treated in order to obtain a multi-phase scaffold based on calcium phosphate (HAp, *β*-TCP, *α*-TCP), calcium silicate (CaSiO_3_, *β*-Ca_2_SiO_4_, Ca_2_SiO_4_) and silicate glass phases. A multi-phase composite strategy is a pathway where advantages of different phosphate- and silicate-based phases are combined to obtain biomaterials with desired properties, such as bioactivity, bioresorption rate and mechanical properties [5]. In addition, bioceramics obtained from biogenic sources are better accepted by the human organism, because of its physicochemical similarity to the mineralogical phase in natural bone tissue [8]. By using biogenic sources, obtained bioceramic materials are substituted with various ions with key role functions for bone metabolism. Several reasons can be listed as advantages of using calcium phosphates as bone graft materials: (i) the main inorganic phase in our bones is apatite; therefore, Ca^2+^ and PO_4_^3−^ ions are present in large quantities in the human body; (ii) various calcium phosphates are resorbed by cell-mediated processes, ensuring precursors for bone resorption and the formation process without the burst and uncontrolled release of a large number of degradation products, (iii) Ca^2+^ and PO_4_^3−^ ions have a direct effect on bone cells, where PO_4_^3−^ ions might be a trigger for an osteoinductive response [30]. Among all calcium phosphates, HAp is mostly studied as an apatite for the main inorganic phase in bones, teeth and enamel. At the physiological pH of 7.2–7.4, the concentration of Ca^2+^ and PO_4_^3−^ ions from calcium phosphates decreases in the order *α*-TCP > OCP > *β*-TCP > HAp [3]. Therefore, with the combination of different calcium phosphates, the bioresorption rate could be adjusted. Although few in vivo studies have reported positive results on *α*-TCP, its resorption rate is too high, and as a result, *α*-TCP has hardly been studied as a single-phase biomaterial [30]. However, in combination with other calcium phosphates with a lower resorption rate, it can lead to biomaterial with unique properties. Further, pure *β*-TCP never occurs in natural bone tissue, while Mg-substituted *β*-TCP (whitlockite, *β*-Ca_9_Mg(HPO_4_)(PO_4_)_6_), observed in obtained scaffolds, occupies 25–35 wt% of the inorganic portion of natural bone tissue [31]. In vivo studies on the bone-regenerative and resorption capacity of porous CaSiO_3_ and *β*-TCP scaffolds were carried out by Xu et al. [32]. Results show that CaSiO_3_ scaffolds have higher bone regeneration and resorption capacity compared to the *β*-TCP scaffold. After 16 weeks in vivo, the area of newly formed bone in the CaSiO_3_ scaffold was 9.56% higher compared to the *β*-TCP scaffold. The in vivo analysis of CaSiO_3_’s regenerative potential evaluated by Xu et al. [32] is in agreement with the results obtained by De Aza et al. [33,34]. It has been suggested that the release of Ca^2+^ ions from calcium silicates leads to the formation of a Si–O− active site on the surface of the scaffold. Further, formed Si–O− active sites promote the nucleation of apatite, while the released Ca^2+^ ion enhances the apatite crystal growth [35,36,37]. Along with formed calcium silicates, the obtained homogeneous glassy Si–O–Si layer ensures additional Si–O− active sites on the scaffolds that led to an increased protein adsorption capacity of the scaffolds. The protein adsorption capacity is an important characteristic of biomaterials as it plays a key role in the nature of the scaffold–tissue interface. Protein adsorption due to body fluid control subsequently controls cell adhesion on the scaffold surface [38].

Understanding the effect of surface properties (e.g., surface potential, roughness and specific surface area) on bone tissue regeneration is key to designing biomaterials for specific applications. Along with surface characteristics, protein adsorption is affected by the pH and ionic composition of the surrounding solution. The implanted scaffold first comes into contact with body fluids, which leads to the quick adsorption of proteins on the surface. Further, the adsorbed protein layer affects cell adhesion, proliferation and differentiation [39]. Since the adsorption of biomolecules on different biomaterials is based on an ion-exchanging process, the obtained results indicate that the obtained Si-doped scaffolds are appropriate for protein adsorption. A biocompatibility test is highly important as a high concentration of dopants can result in cytotoxicity. The MTT assay has been widely used to determine cell viability and potential cytotoxicity of tested biomaterials. Our previous research showed the positive effect of HAp and the PCL-coated HAp scaffolds on cell activity [40]. In this work, cytotoxicity was assessed for CaP_Si scaffolds doped with Si. An MTT assay was performed after 1 and 3 days of hMSC cell culture in DMEM extracts of prepared CaP_Si scaffolds (powder form). Studied CaP_Si scaffolds were non-cytotoxic, and Si doping had a beneficial effect on cell proliferation. However, the osteogenic potential of Si-doped scaffolds needs to be confirmed by using stem cells under static and dynamic conditions. PCL coating on the CaP_Si scaffold was effective at increasing the mechanical properties of scaffolds. Applied vacuum impregnation of the PCL led to filling the crack line defects in CaP_Si scaffolds and inhibited crack propagation. Due to the glassy layer on the surface of CaP_Si scaffolds, the amount of coated PCL decreased with the increasing number of APTES impregnation treatments. The ultimate compressive strength of cortical bone is ~200 MP, and that of trabecular bone is ~8.7 MPa [41]. Due to the lower compressive strength of the obtained PCL/CaP_Si scaffolds of ~1.4 MPa, they can be applied for the repair of small-size and/or non-load-bearing bone defects (e.g., craniomaxillofacial).

## 5. Conclusions

In this study, Si-doped calcium phosphate scaffolds (CaP_Si) were prepared through hydrothermal conversion of cuttlefish bone followed by APTES impregnation–heat treatment cycles. Highly porous CaP_Si scaffolds are composed of multi-phasic calcium phosphates (HAp, *α*-TCP, *β*-TCP), calcium silicates (CaSiO_3_, *β*-Ca_2_SiO_4_, Ca_2_SiO_4_) and silicate glass phases. The highly porous structure of cuttlefish bone was preserved, allowing diffusion of nutrients and metabolic waste through the scaffold volume. The obtained porosity of ~78% and pore size with an average height of 200–300 μm and average width of 40–60 μm are suitable for BTE applications. The cell viability test with hMSC cells indicated no harmful effect of Si doping and confirmed good biocompatibility of investigated CaP_Si scaffolds. The addition of the Si-containing phase (APTES) resulted in the amorphous/glassy surface Si-O-Si layer that led to a decrease in surface roughness and specific surface area and increase in BSA protein adsorption. PCL coating on CaP_Si scaffolds enhanced the mechanical properties of Si-doped scaffolds. The obtained scaffolds can be used for non-load bearing bone defects at the craniomaxillofacial region.

The presented PCL/CaP_Si scaffolds exhibited promising properties for development of artificial bone graft substitutes. Future studies will be focused on the evaluation of the osteogenic potential of prepared Si-doped scaffolds, using stem cells under static and dynamic conditions, through quantitative reverse transcription polymerase chain reaction for osteogenesis-related genes (e.g., alkaline phosphatase, bone sialoprotein, osteocalcin), accompanied by histological (hematoxylin-eosin) and immunohistochemical (collagen type I) staining.

## Figures and Tables

**Table 1:** Quantitative analysis of calcium phosphate and calcium silicate phases in prepared scaffolds performed by whole-powder-pattern decomposition analysis.

	HAp	Wtc	*α*-TCP	CaSiO_3_	*β*-Ca_2_SiO_4_	Ca_2_SiO_4_	CaO	Amor./Glassy Phase
CaP_Si0	73.41	8.11	11.92	-	-	-	0.58	5.98
CaP_Si1	42.94	16.41	3.81	4.76	0.78	2.22	1.10	27.98
CaP_Si2	28.88	21.01	11.17	0.81	2.80	-	-	35.33
CaP_Si3	24.29	24.03	10.11	-	2.64	-	-	38.93
CaP_Si4	24.34	23.71	14.04	-	2.96	-	-	34.95
